# Clinico-pathological significance of immunohistochemically marked tumor-associated macrophage in classic Hodgkin lymphoma

**DOI:** 10.1186/s43046-020-00029-1

**Published:** 2020-04-15

**Authors:** Mona Y. Y. Abd Allah, Maryan Waheeb Fahmi, Shaimaa EL-Ashwah

**Affiliations:** 1 Pathology Department, Faculty of Medicine, Mansoura University, Mansoura, 35516 Egypt; 2 Medical Oncology Internal Medicine Department, Oncology Center, Faculty of Medicine, Mansoura University, Mansoura, 35516 Egypt; 3 Clinical Hematology Internal Medicine Department, Oncology Center, Faculty of Medicine, Mansoura University, Mansoura, 35516 Egypt

**Keywords:** Classic Hodgkin lymphoma (cHL), Tumor-associated macrophage (TAM), CD68, CD163, Disease free survival (DFS), Overall survival (OS)

## Abstract

**Background:**

Tumor-associated macrophages (TAM) are pivotal in remodeling of the tumor immune microenvironment and clinical outcome. Herein, we aim to evaluate the impact of immunohistochemical (IHC) expression of CD68 and CD163 in TAM on clinico-pathological features, patients’ response to therapy and the overall survival (OS).

**Results:**

This retrospective study was performed on paraffin-embedded tissue blocks of 100 classic Hodgkin Lymphoma (cHL) cases diagnosed and treated at our Institution. Immunohistochemical scores of CD68 and CD163 were statistically related to bulky disease (*p* value = 0.005 for both), tumor stage (*p* value = 0.02 for both), International Prognostic Score (IPS) (*p* value = 0.04 and 0.02 respectively), and the overall response rate (ORR) (*p* value = 0.001). Additionally, CD163 was also statistically related to gender (*p* value = 0.02), serum albumin level (*p* value = 0.03), and B symptoms (*p* value = 0.04). HCV seropositivity did not relate to either CD68 or CD163 score. Using univariate analysis revealed that B symptoms, bulky disease, IPS ≥ 3, and CD163 > 25% were associated with lower OS (*p* values = 0.003, 0.006, 0.001, and < 0.001 respectively), while after multivariate cox regression analysis, B symptoms, IPS ≥ 3, and CD163 > 25% were related to inferior OS (*p* values 0.02, 0.02, and 0.003).

**Conclusion:**

CD163 expressing TAM is a powerful predictor for OS in cHL, unlike CD68.

## Background

Hodgkin lymphoma (HL) is a unique neoplasm of B cell that represents about 10% of all diagnosed lymphomas in the USA [1]. The crude incidence of HL in the European Union represents about 2.3, and its mortality rate is about 0.4 cases/100.000/year [2]. In Egypt, the incidence of HL is 1.5 per 100.000 populations [3].

Classic Hodgkin’s Lymphoma (cHL) represents about 95% of all cases of HL and is characterized by the presence of Hodgkin Reed-Sternberg cells (HRS) with many reactive cells in tumor microenvironment such as macrophages, lymphocytes, neutrophils, histiocytes, eosinophils, and plasma cells [2].

cHL microenvironment is unique as the tumor cells (HRS) normally constitute less than 1% of the tumor cellularity and are surrounded by an abundant and heterogeneous inflammatory infiltrate. The interaction between HRS and the microenvironment reactive cells sustain tumor growth and survival [4]. The constituent cells of this microenvironment may expect the clinical outcomes in cHL [5, 6].

Although, it is a potentially curable disease, about 20% of patients still die from progressive disease and another proportion may be over treated leading to solid tumors and end organ dysfunction as sequel [7]. Thus, it is mandatory to predict those cases with a likelihood of treatment failure at the time of diagnosis. International Prognostic Score (IPS) is the most commonly used prognostic stratification system, although, this model is less suitable for patients with limited stage disease and it fails to identify a group of patients whose probability of cure is less than 50% [8].

A recent meta-analysis found that tumor-associated macrophages (TAM) which were identified by immunohistochemistry (IHC), CD68 or CD163, in cHL microenvironment could be a predictor of adverse outcome, but this result was limited by poor accessibility to negative studies and variation in the included studies regarding follow-up duration and endpoints [9]. Another large study was carried on 265 patients concluded no association between TAM and prognosis of cHL [10].

Regarding the debatable prognostic role of TAM in cHL, we evaluated in this study the IHC staining for both CD68 and CD163 to assess the TAM in Egyptian patients diagnosed with cHL. Also, the relation of TAM with the clinico-pathological characteristic, patients’ response to therapy and their impact on patients’ outcome and survival were studied.

## Methods

### Patients and clinico-pathological characteristics

We performed a retrospective study on formalin-fixed paraffin-embedded tissue blocks of 100 cases diagnosed with cHL which were collected from the archive of pathology laboratory at our institution from 2008 to 2016 where the patients were diagnosed according to the results of the routine morphological and immunohistochemical examination of lymph node biopsy materials. The histopathological classification for all studied cases was made according to the World Health Organization (WHO) criteria. All included patients were subjected to comprehensive clinical history, physical examination, CT scan, and laboratory and radiological investigations. Patients were staged according to the Ann Arbor staging system and were categorized into stages I, II, III, and IV. Relying upon several risk factors including patient’s age, gender, stage, and presence of hypoalbuminemia and lymphopenia, the International Prognostic Score (IPS) was categorized as: low-risk IPS if presented with up to two risk factors and high-risk IPS if presented with three or more risk factors [11].

Most patients (90%) were treated with adriamycin, bleomycin, vinblastine, and dacarbazine (ABVD) protocol, and only 10 patients (10%) were treated with combined modality as 1st line therapy. The attained response was defined as complete remission (CR) when the patient became completely free from nodal enlargement by CT or PET/CT scans as well as normalization of the initially abnormal laboratory tests and/or biopsies for at least 1 month. On the other hand, partial response (PR) was defined as there was remaining nodal enlargement which is decreased in size and number by at least 50%. Relapse was considered when there was appearance of new lymph nodes after previous remission, either complete or partial response to therapy. This is confirmed by abnormal hilum by CT and/or lymphoma infiltration with biopsy, or only bone marrow involvement without nodal enlargement.

Recorded follow-up data was included to define the disease-free survival (DFS) as the time between the date of CR to time of progression, relapse, or death, while the overall survival (OS) was recognized as the time between date of diagnosis to the date of death from any reasons or last date of follow-up for still alive patients.

The exclusion criteria include:

1. Patients with previous history of any other malignancy,
2. Relapsed or refractory cHL and
3. Insufficient recorded laboratory, radiological, follow-up data or paraffin blocks.

The current study was reviewed and approved by our Institutional review board (IRB) of our faculty of medicine (Code number: R/18.02.27).

### Immunohistochemistry

Immunohistochemistry (IHC) for CD68 and CD163 were done according to the manufacturer’s data sheet. Paraffin blocks of the selected cases were sectioned 4-μm thick, deparaffinized, and rehydrated then incubated for about 30 min with 0.3% hydrogen peroxide in methanol to block endogenous peroxidase activity followed by epitope retrieval using heat-induced epitope retrieval (HIER) for 30 min in EDTA buffer solution, pH 8.0 together with a pressure cooker followed by rinsing in distilled water and phosphate-buffered saline (PBS). The prepared tissue sections were then incubated for 30 min at room temperature with primary antibodies against monoclonal mouse CD68/Macrophage Marker Ab-3 Antibody (catalog #: MS-397-R7 [7.0 ml], Thermo Fisher Scientific, UK) and monoclonal mouse CD163 Antibody (catalog #: MS-1103-S0,-S1 or -S [0.1 ml, 0.5 ml, or 1.0 ml supernatant], Thermo Fisher Scientific, UK). Immunoperoxidase method was done using ImmunoPure Ultra-Sensitive ABC Peroxidase (catalog #: TL-060-PHJ, Thermo-scientific, UK), using diaminobenzidine as chromogen (catalog #: TA-125-HDX, Thermo-scientific, UK). After that, tissue sections were washed in distilled water then counterstained with hematoxylin followed by dehydration in ascending grades of alcohol and xylene and finally covered with coverslips.

### Evaluation of the IHC

Both CD68 and CD163 expressions were semi-quantitatively assessed by the pathologist for each case. The percentage of positive cells was considered in the evaluation.

Both CD68 and CD163 positive macrophage showed membrane-associated reactivity. The percentage of CD68 and CD163 positive macrophages was determined in relation to negative Reed-Sternberg cells and reactive inflammatory cells in the background. The staining was detected in the micro-environment in Reed-Sternberg rich areas and was evaluated regardless if it is a hot-spot or not and scored as: I (< 5%), II (5–25%), and III (> 25%) [12]. For quality control, an internal positive control (the endothelial cells lining the adjacent vascular spaces) as well as negative control (slides which were not incubated by primary antibodies) were used.

### Statistical analysis

Data were tabulated then statistically analyzed using Statistical Package for Social Sciences (SPSS), version 22. χ2 (Chi-square) or Fisher exact tests were used to test significant differences between groups. Chi-Square (χ2) test was used for comparison of 2 or more groups. Fischer Exact test was used as correction for Chi-Square test when more than 25% of cells have count less than 5 in 2 × 2 tables.

The survival curves were performed using Kaplan Meier method to detect mean and median survival times. The differences between the curves were studied using the log-rank test. Multivariate Cox regression was used to identify predictors and risk factors affecting the survival. In all tests, *P* ≤ 0.05 was considered to be statistically significant.

## Results

### Clinico-pathological and histological features of the studied cases (Table 1)

This study included 100 patients diagnosed with cHL. Their median age was 33 (ranged 15–74 years). The male:female ratio was 37%:63%. The hemoglobin level was less than 10.5 g/dl in 36 patients (36%). The white blood cell count was ≥ 15 × 109/l in 15% of patients, and 35 patients (35%) had less than 4 g/dl serum albumin levels. Screening for HIV antibodies, HBs Ag, and HCV antibodies is routinely carried out for patients under treatment in our oncology center unlike, EBV status which was not investigated. No included patients were positive for HIV antibodies or HBs Ag, but HCV antibodies were detected in sera of 14 patients (14%). Ninety-five patients had performance status (0 and 1). The majority of patients (54%) exhibited B symptoms, and 11 patients (11%) presented with bulky disease.

**Table 1:** Clinico-pathological characteristics of the included 100 patients

Item	Value	Percentage
Age (median)	33 (15–74) years	
Gender	Male: female	37%: 63%
HB level	≥ 10.5 g/dl	64%
	< 10.5 g/dl	36%
WBC	≥ 15 × 10^9^/l	15%
	< 15 × 10^9^/l	85%
Serum albumin	<4 g/dl	35%
	≥4 g/dl	65%
HCV antibodies	Positive	14%
	Negative	86%
Performance status	<2	95%
	≥2	5%
B symptoms	Absent	46%
	Present	54%
Bulky disease	No	89%
	Yes	11%
Histopathology subtype	Mixed cellularity	46%
	Nodular sclerosis	42%
	Lymphocyte rich	12%
Extra nodal disease	No	73%
	Yes	27%
Spleen involvement	No	78%
	Yes	22%
Bone marrow involvement	No	96%
	Yes	4%
Ann Arbor stage	I	1%
	II	27%
	III	42%
	IV	30%
Stage	Limited	21%
	Advanced	79%
IPS	< 3	60%
	≥ 3	40%
CD 68	I	41%
	II	41%
	III	18%
CD 163	I	37%
	II	40%
	III	23%
Response	CR	61%
	PR	12%
	PD	7%
	SD	20%
Relapse after CR	Yes:no	20%:41%
Status	Alive:dead	82%:18%

*HB* hemoglobin, *WBC* white blood cell count, *HCV*, hepatitis C virus, *CR* complete response, *PR* partial Response, *PD* progressive disease, *SD* stationary disease, *IPS* International Prognostic score

Most of the patients had histological features consistent with mixed cellularity subtype (46% of patients) followed by nodular sclerosis 42% while the least in incidence was lymphocyte rich 12%.

Twenty-seven patients (27%) had extra nodal disease; splenic involvement was detected in 22% of cases while the bone marrow was infiltrated only in 4 patients (4%). Using Ann Arbor stage, one patient (1%) was diagnosed as stage I, 27 patients (27%) were stage II, 42 patients (42%) were stage III, and 30 patients (30%) were stage IV. Limited stage was detected in 21 out of 100 patients (21%) whereas 79% of patients were advanced stage. Calculated IPS for included patients was more than or equal to 3 in 40 patients (40%).

Ninety patients (90%) were treated with ABVD protocol, and 10 patients (10%) were treated with combined modality ABVD + IFRT as 1st line therapy; 61 out of 100 patients (61%) showed CR, whereas 12 patients (12%) revealed PR, 20 patients (20%) showed stable disease (SD), and 7 patients (7%) revealed progressive disease (PD). Twenty patients (20%) relapsed after achieving 1st CR. Fifty-four patients (54%) received 2nd line chemotherapy. The follow-up duration ranged from 0.29 to 10.2 years. Eighty-two patients (82%) were still alive so the median OS was not reached.

### Relation of CD68 and CD163 expression with clinico-pathological parameters

**Fig. 1:**
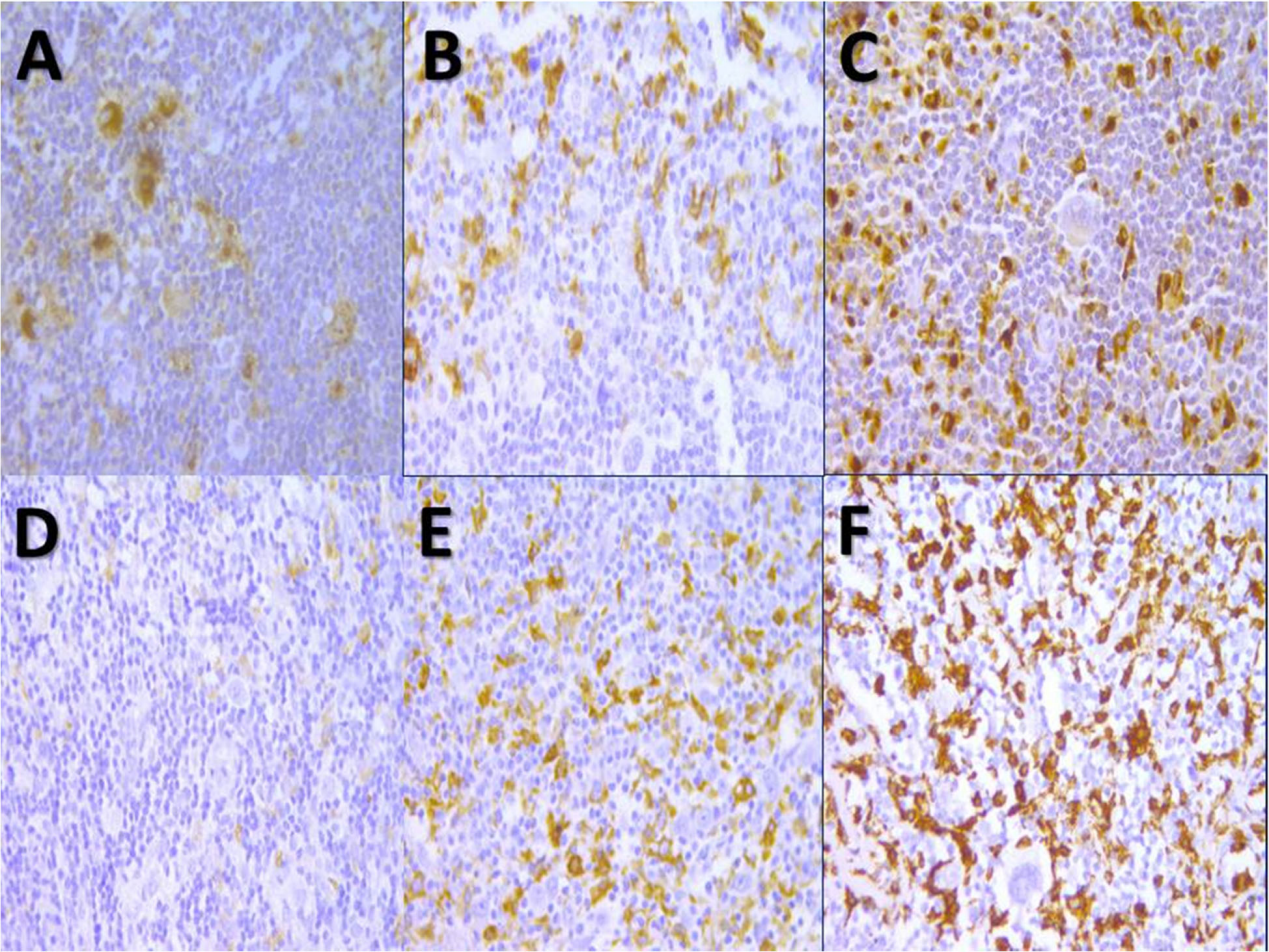
Immunohistochemical expression of CD68 and CD163 (× 400). **a** CD68 immunoreactivity in < 5% of cells (score I), **b** CD68 immunoreactivity in 5–25% of cells (score II), **c** CD68 immunoreactivity in > 25% of cells (score III), **d** CD163 immunoreactivity in < 5% of cells (score I), **e** CD163 immunoreactivity in 5–25% of cells (score II), and **f** CD163 immunoreactivity in > 25% of cells (score III)

Using IHC staining for CD68, 41 patients had score I (< 5%), 41 patients had score II (5–25%), and 18 patients had score III (> 25%) (Fig. 1a, b, and c). CD68 score was statistically related to bulky disease (*p* = 0.005), stage (*p* = 0.02), IPS (*p* 0.04), and the overall response rate (ORR) (*p* = 0.001), so that higher CD68 score was statistically related to the presence of bulky disease, advanced stage, IPS score, and lower ORR. On the other hand, there was no statistically significant difference between IHC expression of CD68 and patient’s age, gender, hemoglobin, WBC, albumin levels, presence or absence of B symptoms, histopathology subtype, and presence or absence of extra nodal disease as illustrated in Table 2.

**Table 2:** Clinico-pathological characteristics in relation to IHC expression of CD68 and CD163

		CD68			*P* value	CD163			*P* value
		1	2	3		1	2	3	
Age category	< 45 years	31%	30%	11%	0.5	29%	31%	12%	0.05*
	≥ 45 years	10%	11%	7%		8%	9%	11%	
Gender	Male	10%	19%	8%	0.5	9%	14%	14%	0.02*
	Female	31%	22%	10%		28%	26%	9%	
HB category	< 10.5 g/dl	17%	13%	6%	0.6	16%	30%	10%	0.17
	≥ 10.5 g/dl	24%	28%	12%		21%	27%	13%	
WBCs category	< 15 × 10^9^/l	35%	34%	16%	0.8	30%	35%	20%	0.7
	≥ 15× 10^9^/l	6%	7%	2%		7%	5%	3%	
Albumin level	< 4 g/dl	16%	14%	5%	0.9	15%	10%	10%	0.03*
	≥ 4 g/dl	24%	21%	7%					
						20%	27%	5%	
HCV virology	Negative	35%	34%	14%	0.4	32%	34%	20%	0.9
	Positive	6%	7%	1%		5%	6%	3%	
B symptoms	Absent	23%	15%	8%	0.2	23%	16%	7%	0.04*
	Present	18%	16%	10%		14%	24%	16%	
Bulky disease	No	40%	37%	12%	0.005*	36%	37%	16%	0.005*
	Yes	1%	4%	6%		1%	3%	7%	
Histopathology subtype	LR	4%	7%	1%	0.5	5%	4%	3%	0.86
	MC	21%	18%	7%		16%	21%	9%	
	NS	16%	16%	10%		16%	15%	11%	
Extra nodal disease	No	34%	25%	14%	0.07	29%	27%	17%	0.55
	Yes	7%	16%	4%		8%	13%	6%	
Stage	Limited	14%	4%	3%	0.02*	13%	6%	2%	0.02*
	Advanced	27%	37%	15%		24%	34%	21%	
IPS	< 3	30%	23%	7%	0.04*	25%	27%	8%	0.02*
	≥ 3	11%	18%	11%		12%	13%	15%	
ORR	CR/PR	38%	24%	11%	0.001*	34%	28%	11%	0.001*
	NO CR/PR	3%	17%	7%		3%	12%	12%	

*HB* hemoglobin, *WBC* white blood cell count, *HCV* Hepatitis C virus, *MC* mixed cellularity, *NS* nodular sclerosis, *LR* lymphocyte rich, *IPS* International Prognostic score, *ORR* overall response rate, *CR* complete response, *PR* partial response

Additionally, using IHC staining for CD163, 37 patients had score I, 40 patients had score II, and 23 patients had score III (Fig. 1d, e, and f). CD163 score was statistically correlated to patient’s age (*p* = 0.05), gender (*p* = 0.02), serum albumin level (*p* = 0.03), B symptoms (*p* = 0.04), bulky disease (*p* = 0.005), stage (*p* = 0.02), IPS (*p* 0.02) and ORR (*p* = 0.001), such that higher CD163 score was statistically related to older age, male gender, lower serum albumin, presence of B symptoms, bulky disease, advanced stage, and lower ORR. But there was statistically insignificant difference between IHC expression of CD163 and hemoglobin categories, WBCs categories, histopathology subtype, and presence or absence of extra nodal disease as illustrated in Table 2.

### Relation of CD68 and CD163 to OS

**Fig. 2:**
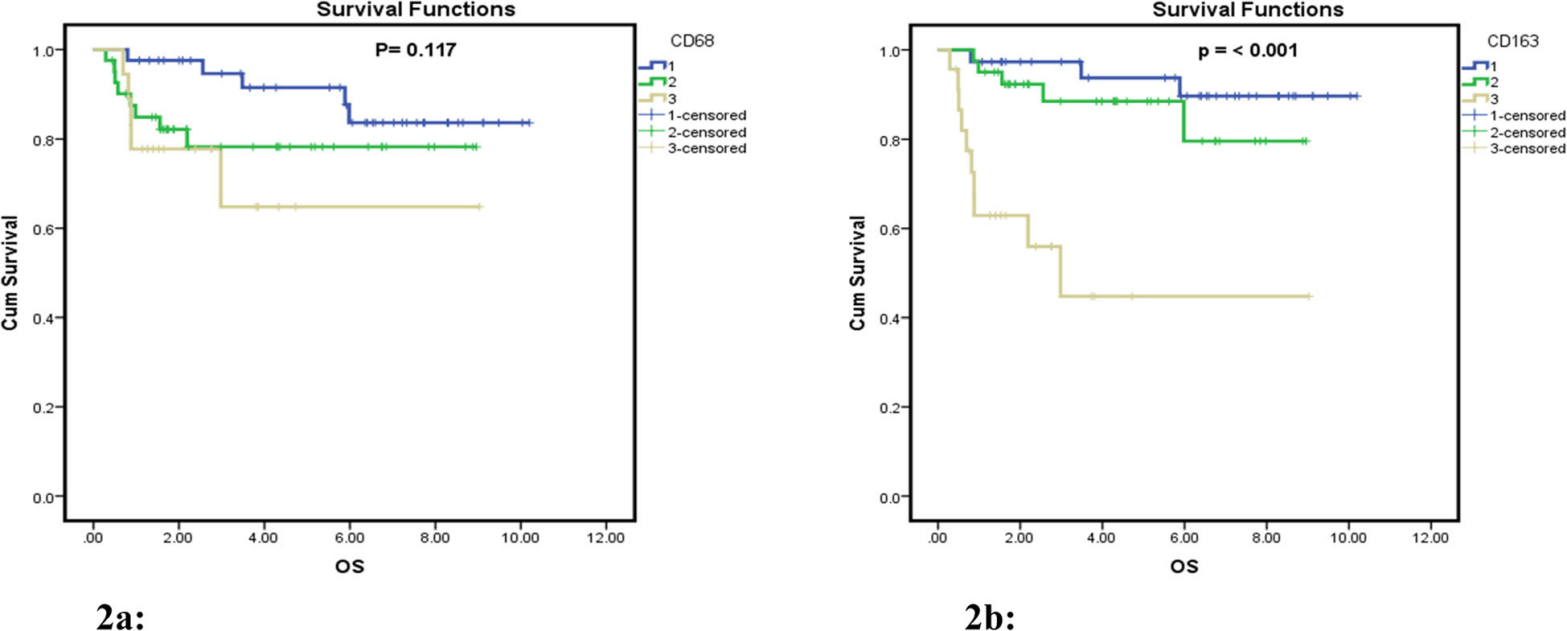
**a** Kaplan Meier curve for overall patients’ survival. It revealed statistically insignificant decrease in OS with increasing CD68 expression. **b** Kaplan Meier curve for overall patients’ survival. It revealed statistically significant decrease in OS with increasing CD163 expression (inverse relationship)

Kaplan Meier curve for patients’ overall survival (OS) revealed decrease in OS insignificantly with increasing CD68 and significantly with increasing CD163 expression (inverse relationship) (Fig. 2a, b). The univariate analysis of OS showed that OS was statistically affected by B symptoms (*p* = 0.003), bulky disease (*p* = 0.006), IPS (*p* = 0.001), and CD163 score (*p* = <0.001) but not affected by CD68 score (*p* = 0.1) as illustrated in Table 3.

**Table 3:** Univariate analysis of factors affecting OS and DFS

		OS			DFS		
		Median	Log rank test	*P* value	Median	Log rank test	*P* value
B symptoms	Absent	9.587	8.61	0.003*	5.822	0.459	0.49
	Present	7.216			5.626		
Bulky lesion	No	8.726	7.66	0.006*	6.348	3.9	0.05*
					2.342		
	Yes	5.073					
IPS	< 3	9.36	11.24	0.001*	6.18	0.14	0.7
	≥ 3	6.02			5.77		
CD68	1	9.198	4.28	0.1	7.084	13.07	0.001*
	2	7.235			4.876		
	3	6.422			1.801		
CD 163	1	9.526	25.3	< 0.001**	7.216	27.7	<0.001**
	2	7.849			5.059		
	3	4.768			1.305		

*OS* overall survival, *DFS* disease-free survival, *IPS* International Prognostic score

As no consensus on optimal cutoff of CD68 and CD163 is well recognized, we studied impact of CD163 at cutoff > 5% (score 2) as well as CD163 at cutoff > 25% (score 3) on OS in multivariate analysis. This multivariate cox regression analysis for OS revealed that B symptoms (*p* = 0.024), IPS ≥ 3 (*p* = 0.023), and CD163 at cutoff > 25% (score 3) (*p* = 0.003) statistically affected OS while, CD163 at cutoff > 5% (score 2) did not affect OS (*p* value 0.44) (as illustrated in Table 4).

**Table 4:** Multivariate analysis of factors affecting OS and DFS

	OS				DFS		
	HR	95.0% CI	*P*		HR	95.0% CI	*P*
B symptoms	4.55	1.21–16.96	0.02*	**Bulky disease**	1.22	0.24-6.06	0.8
Bulky disease	0.66	0.2–2.18	0.5	**CD68**	0.88		
IPS ≥ 3	3.55	1.95–10.521	0.02*	**CD68 (score II)**	0.83	0.12–5.8	0.85
CD163	0.003*			**CD68 (score III)**	0.36	0.007–19.6	0.62
CD163 (score II)	1.78	0.41–7.74	0.44	**CD163**	0.3		
CD163 (score III)	9.39	2.19–40.22	0.003*	**CD163 (score II)**	2.0	0.3–13.1	0.47
				**CD163 (score III)**	25.5	0.41–1571.7	0.12

*OS* overall survival, *DFS* disease-free survival, *HR* hazard ratio, *CI* confidence interval, *IPS* International Prognostic score

### Relation of CD68 and CD163 to DFS

**Fig. 3:**
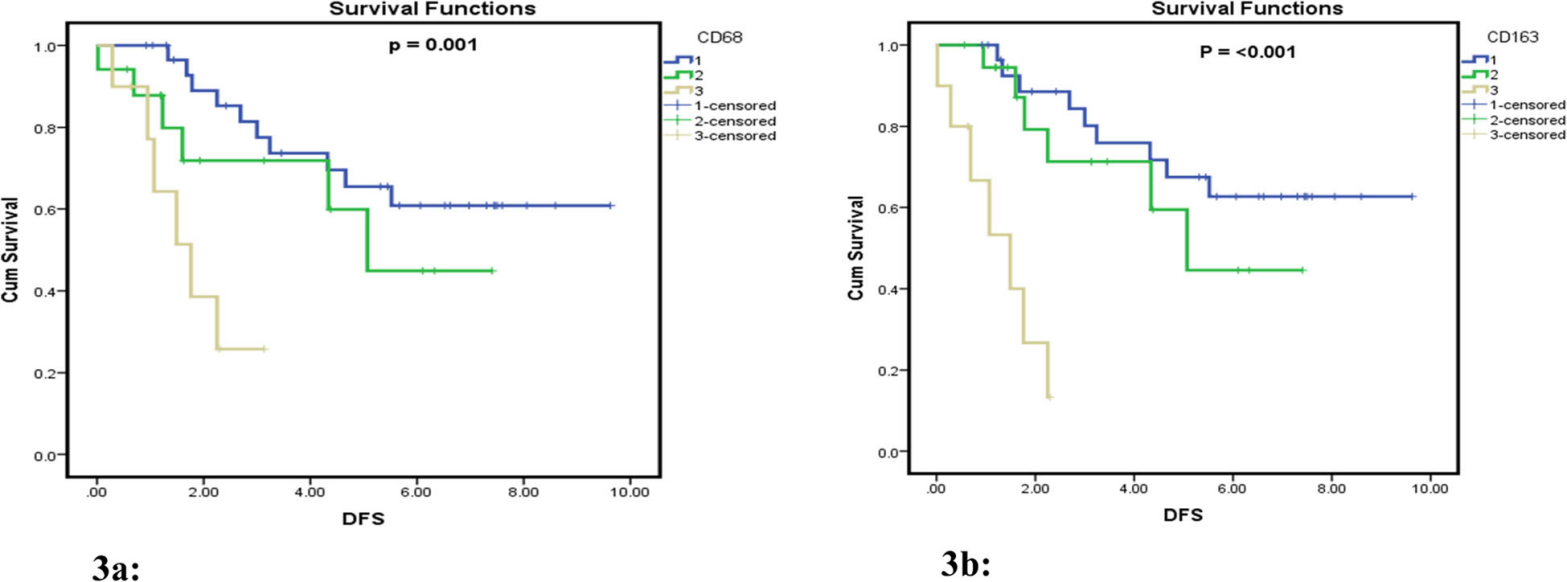
**a** Kaplan Meier curve for disease-free survival. It revealed statistically significant decrease in DFS with increasing CD68 expression (inverse relationship). **b** Kaplan Meier curve for disease-free survival. It revealed statistically significant decrease in DFS with increasing CD163 expression (inverse relationship)

Kaplan Meier curve for the disease-free survival (DFS) revealed significant decrease in DFS with increasing both CD68 and CD163 expression (inverse relationship) (Fig. 3a, b).

The univariate analysis of DFS demonstrated that DFS was affected by bulky disease (*p* = 0.05), CD68 score (*p* = 0.001), and CD163 score (*p* = 0.001) as illustrated in Table 3.

Multivariate Cox regression analysis was applied to study factors affecting DFS including both of CD68 and CD133 at cutoff > 5% (score 2) and at cutoff > 25% (score 3).This multivariate analysis revealed that neither bulky disease, nor CD68 or CD163 (at either cutoff values) statistically affected DFS as illustrated in Table 4.

## Discussion

Macrophages are heterogeneous cells which are pivotal in remodeling of the tumor immune microenvironment. Relation between TAM and outcome of several malignancies attracted many researchers [13].

CD68 is recognized as a pan-macrophage biomarker and also expressed by other cells as monocytes, some subtypes of CD34-positive hematopoietic stem cells (myeloid cells), fibroblasts, dendritic cells, neutrophils, langerhans cells, basophils, and mast cells [14]. It is therefore non-specific for the monocyte/macrophage lineage.

On the other hand, expression of CD163 is largely restricted to a subdivision of macrophage known as M2 macrophages. These M2 macrophages are involved in anti-inflammatory function. They may be related to cell proliferation and migration. They may also play an important role in new blood vessel formation by regulating the angiogenic switch via secretion of vascular endothelial growth factor (VEGF) and hypoxia-inducing factors (HIF). M2 macrophages, therefore, may induce tumor growth and metastasis unlike M1 macrophages that kill tumor cells [13, 15, 16].

Therefore, unlike CD68, CD163 staining pattern is reported to show a cleaner background with less non-specific staining of RS cells and other inflammatory cells. Thus, CD163 expression was easier to evaluate than CD68 expression.

This work spots light on the possible role of cHL microenvironment in disease biological behavior using IHC assessment of CD68 and CD163 expression in TAM of 100 cases diagnosed as cHL and study its relation to different clinico-pathological parameters, response to treatment, and overall survival.

In this study, we noticed that CD68 and CD163 scores correlated statistically to tumor stage (*p* value = 0.02 for both), bulky disease (*p* value = 0.005 for both) and IPS (*p* value = 0.04 and 0.02 respectively). Additionally, CD163 score was also related to gender (*p* value = 0.02), serum albumin (*p* value = 0.03), B symptoms (*p* value = 0.04). This agrees with Guo et al. who revealed that CD68 or CD163 positive TAMs were associated with advanced tumor stage, presence of bulky disease, higher IPS, and presence of B symptoms after meta-analysis of 22 studies [9].

On the other hand, Azambuja et al. found no association between various clinico-pathological characteristics and the expression of either CD68 or CD163 [10]. This discrepancy between our results and that of Azambuja et al. could be attributed to different methods of IHC interpretation, in addition to different number of cases in each study.

Also, our results elicited that higher scores of CD68 and CD163 were associated with lower ORR (*p* value = 0.001); this copes with many previous studies that found TAMs enhance the resistance to curative chemotherapy and radiotherapy in many malignancies [13, 17, 18].

For impact of TAM on prognosis, as regard DFS, we found higher CD68 and CD163 scores were associated with lower DFS (*p* value = 0.001 and < 0.001 respectively) using univariate analysis but neither CD68 nor CD163 statistically affected DFS after applying multivariate analysis. Furthermore, regarding OS, multivariate Cox regression analysis identified that the presence of B symptoms (*p* value = 0.02), IPS ≥ 3 (*p* value = 0.02), and high CD163 score (> 25%) (*P* value = 0.003) as variables statistically associated with lower OS but not CD68 score. This gives advantage for CD163 over CD68 in prediction of poor overall survival. Our results agree with Klein et al. who identified CD163 score ≥ 25% and IPS ≥ 4 as predictors of poor survival after assessment of CD68 and CD163 expression by five hematopathologists [19]. Also, Guo et al. noted that high expression of CD163 and CD68 in TAM was related to poor prognosis [9]. On the contrary, Azambuja et al. showed no association between CD68 and CD163 in TAM and patients’ outcome and prognosis.

Discrepancies among studies could be attributed in part to the different number of cases in each study and in other part to the different assessment of low scores (5 to 25%) of CD68 due to the non-specific reactivity of RS cells as well as the reactive inflammatory cells in the background.

## Conclusion

Increased expression of CD68 and CD163 in TAM were associated with adverse clinico-pathological features including bulky lesion, advanced tumor stage, high IPS ≥ 3 and lower response rate; however, CD163 is an outstanding predictor for OS in cHL rather than CD68. Thus, new therapeutic strategies altering cHL microenvironment may be helpful in patients with conventional therapy failure.

## Data Availability

All the clinical, radiological, and pathological data used in this manuscript is available on Mansoura University medical system (Ibn Sina Hospital management system). http://srv137.mans.edu.eg/mus/newSystem/ IHC results for CD68 and CD163 are available from Associate Professor of Pathology Dr. Mona Y.Y. Abd Allah on reasonable request.

## Abbreviations

ABVD: Adriamycin, bleomycin, vinblastine, and dacarbazine
cHL: Classic Hodgkin Lymphoma
CR: Complete remission
DFS: Disease-free survival
EDTA: Ethylenediaminetetraacetic acid
HBs Ag: Hepatitis B surface antigen
HCV: Hepatitis C virus
HIV: Human immunodeficiency virus
HIER: Heat-induced epitope retrieval
HIF: Hypoxia inducing factor
HL: Hodgkin lymphoma
HRS: Hodgkin Reed-Sternberg cells
IFRT: Involved-field radiotherapy
IHC: Immunohistochemistry
IPS: International Prognostic Score
IRB: Institutional review board
ORR: Overall response rate
OS: Overall survival
PBS: Phosphate-buffered saline
PD: Progressive disease
PET/CT scan: Positron emission tomography/computerized tomography scan
PR: Partial response
SD: Stable disease
SPSS: Statistical Package for Social Sciences
TAM: Tumor-associated macrophages
VEGF: Vascular endothelial growth factor
WHO: World Health Organization
